# Efficacy of *Boesenbergia rotunda* Treatment against Thioacetamide-Induced Liver Cirrhosis in a Rat Model

**DOI:** 10.1155/2012/137083

**Published:** 2012-09-04

**Authors:** Suzy M. Salama, Mehmet Bilgen, Ahmed S. Al Rashdi, Mahmood A. Abdulla

**Affiliations:** ^1^Department of Molecular Medicine, Faculty of Medicine, University Malaya, 50603 Kuala Lumpur, Malaysia; ^2^Radiology Department, Faculty of Medicine, Erciyes University, Kayseri 38039, Turkey

## Abstract

*Background*. Experimental research in hepatology has focused on developing traditional medicines into potential pharmacological solutions aimed at protecting liver from cirrhosis. Along the same line, this study investigated the effects of ethanol-based extract from a traditional medicine plant *Boesenbergia rotunda* (BR) on liver cirrhosis. *Methodology/Results*. The BR extract was tested for toxicity on 3 groups of rats subjected to vehicle (10% Tween 20, 5 mL/kg) and 2g/kg and 5g/kg doses of the extract, respectively. Next, experiments were conducted on a rat model of cirrhosis induced by thioacetamide injection. The rats were divided into five groups and, respectively, administered orally with 10% Tween-20 (5 mL/kg) (normal control group), 10% Tween-20 (5 mL/kg) (cirrhosis control group), 50 mg/kg of silymarin (reference control group), and 250 mg/kg and 500 mg/kg of BR extract (experimental groups) daily for 8 weeks. The rats in normal group were intraperitoneally injected with sterile distilled water (1 mL/kg) 3 times/week, and those in the remaining groups were injected intraperitoneally with thioacetamide (200 mg/kg) thrice weekly. At the end of the 8 weeks, the animals were sacrificed and samples were collected for comprehensive histopathological, coagulation profile and biochemical evaluations. Also, the antioxidant activity of the BR extract was determined and compared with that of silymarin. Data from the acute toxicity tests showed that the extract was safe to use. Histological analysis of the livers of the rats in cirrhosis control group revealed uniform coarse granules on their surfaces, hepatocytic necrosis, and lymphocytes infiltration. But, the surfaces morphologically looked much smoother and the cell damage was much lesser in those livers from the normal control, silymarin and BR-treated groups. In the high-dose BR treatment group, the livers of the rats exhibited nearly normal looking lobular architecture, minimal inflammation, and minimal hepatocyte damage, the levels of the serum biomarkers and liver enzymes read nearly normal, and these results were all comparable to those observed or quantified from the normal and silymarin-treated groups. The BR extract had the antioxidant activity about half of what was recorded for silymarin. *Conclusion*. The progression of the liver cirrhosis can be intervened using the ethanol-based BR extract, and the liver's status quo of property, structure, and function can be preserved. This capability of the extract warrants further studies exploring the significance of its pharmacologic potential in successfully treating the liver cirrhosis in humans.

## 1. Introduction

Pharmaceutical compounds with formulations based on interferon, colchicines, penicillamine, and corticosteroids are currently available for treating common liver diseases of cirrhosis, fatty liver, and chronic hepatitis, but with either inconsistent efficacies or high incidences of side effects [1]. A number of natural compounds extracted from plants offer alternative treatment options that are safe and effective [2]. Extracts from newly discovered or already known plant species are constantly being tested on animal model systems mimicking human diseases and injuries [3]. Presently, many natural extracts are used for treating human disorders in organs, but other than the liver [4]. Therefore, the potential roles and effectiveness of these extracts in liver diseases are yet to be studied. An extract obtained from the perennial herb *Boesenbergia rotunda* (BR) is one of those in this category and waiting for exploration of its role in liver pathologies.

The plant BR belongs to the family Zingiberaceae and is traditionally called *temu kunci*. With unique finger-like rhizomes, it is commonly used as a folk medicine in Southeast Asia for treating several diseases including aphthous, dry mouth, stomach discomfort, leucorrhea, and dysentery. Scientific investigations in the past have reported that the extracts isolated from the BR plant using various solutions (such as methanol, hexane, or chloroform) have neuroprotective [5], antibacterial [6], anticancer [7], antifeedant [8], and antiviral [9] effects. The methanol-based extract was shown to contain chemical compounds Quercetin and Kaempferol, which are known to play critical role in antioxidant and anti-inflammatory cascades or processes [10]. When the hexane or chloroform is used in the isolation process, the resulting extract contains other important anti-oxidants: three flavanones (pinostrobin, pinocembrin, and alpinetin) and two chalcones (cardamonin and boesenbergin) [11].

Our laboratory has been investigating the therapeutic values of various plant-based extracts in prevention and protection of liver against toxins [12]. As a continuation of our efforts, in this study, for the first time, we evaluated the previously demonstrated anti-oxidant property of the ethanol-based BR extract in progressive liver damage. In particular, we tested the efficacy of the extract as a therapeutic agent on a rat model of liver cirrhosis induced chemically by thioacetamide (TAA) administration. We have been working with this experimental model because it closely mimics the etiology and pathology of the disease seen in humans.

To objectively evaluate the therapeutic value of the BR extract on the liver cirrhosis, we also employed another herbal substance silymarin—a hepatoprotectant with a well-established record [13]. Silymarin is a purified extract obtained from the seeds of the plant *Silybum marianum* and used widely as a supportive therapy for liver disorders such as cirrhosis, hepatitis, and fatty acid infiltration due to alcohol and toxic chemicals [14]. We compared the positive effects achieved with the BR extract on the cirrhotic liver against the benchmark protection provided by silymarin. In the following, we describe each of the processes and procedures employed in our experiments for assessing the anti-oxidant power, toxicity, and effectiveness of the BR extract. We present extensive data showing pathological and biochemical changes obtained with and without the extract treatment in groups of experiments and discuss our findings in detail regarding the merit of theBR extract as a potential agent for protecting the liver from cirrhosis.

## 2. Materials and Methods

### 2.1. Experimental Animals

Animal protocols governing the experiments were approved by the Ethics Committee for Animal Experimentation, Faculty of Medicine, University of Malaya, Malaysia and the Ethic number PM/07/05/2010/MMA (a) (R) and PM/28/08/2010/MAA (R). Sprague Dawley rats of 6–8 weeks old and weighed between 180 and 200 g were obtained from the institutional animal facility. Throughout the study, the rats were cared humanely and maintained for their normal circadian rhythms by following the guidelines provided in the “Guide for the Care and Use of laboratory Animals” which was prepared by the National Academy of Sciences and published by the National Institute of Health, Malaysia. The rats were given standard pellet diet and tap water, kept in wire-bottomed cages at 25 ± 2°C, exposed to 12 hours light and dark cycle, and housed in an animal room with 50–60% humidity range.

The study was performed in three phases. The first phase involved removing the extract from the BR plant rhizomes and measuring its anti-oxidative property. In the second phase, the toxicity of the extract was examined on 36 (18 males and 18 females) healthy *Sprague Dawley* rats. In the third phase, the efficacy of the extract on inhibiting the development of liver cirrhosis was evaluated using 30 healthy adult male *Sprague Dawley* rats weighing 200–240 g. This experimental phase required chemically inducing cirrhosis by TAA injection to the rats and also using another plant extract silymarin for a reference comparison.

### 2.2. Extract Removal from the Plant BR

Fresh rhizomes of the plant BR were purchased from a commercial company (Ethno Resources Sdn Bhd, Selangor, Malaysia), and identified by comparing it with the voucher specimen deposited at the Herbarium of Rimba Ilmu, Institute of Science Biology, University of Malaya, Kuala Lumpur, Malaysia. After washing with tap water first and then distilled water later, the rhizomes were sliced and left in a shade for a duration of 10 days to dry out. The dried samples were then grounded finely, and 100 g of the resulting powder was mixed in 1000 mL solution of 95% ethanol for 7 days at room temperature. The ethanol extract was distilled under a reduced pressure in Eyela Rotary Evaporator (Sigma-Aldrich, USA), and dried at 40°C in an incubator for 3 days giving a gummy yield of 9.49% (w/w). For the oral administration to the rats, the final product was further dissolved in Tween 20 (10% w/v) and the desired dose for the administration was expressed as concentration in mg/mL per body weight in kg.

### 2.3. Antioxidant Power of the BR Extract

The anti-oxidant power of the BR extract was determined using a test sensitive to its scavenging ability towards reactive oxygen species or reagents containing iron. In this regard, the ferric reducing anti-oxidant power (FRAP) of the BR extract was determined using an assay by following the method described in [15], but with a slight modification. The FRAP reagent was prepared by mixing 300 mM acetate buffer (3.1 mg sodium acetate/mL, pH 3.6), 10 mM 2,4,6-tripyridyl-S-triazine (TPTZ) (Merck, Darmstadt Germany) solution and 20 mM FeCl_3_·H_2_O (5.4 mg/mL). The BR extract and the following standards: Gallic acid, Quercetin, Ascorbic acid, Rutin, Trolox, and 2,6-di-tert-butyl-4-methyl phenol (BHT), were sampled in amounts of 10 *μ*L of 1 mg/mL and 10 *μ*L each along with 10 *μ*L of 0.1 mg/mL silymarin. To each sample separately, 290 *μ*L of the reagent TPTZ were added. The absorbencies of the resulting mixtures were read repetitively at every 4 min for up to 2 hr using ELISA reader (Shimadzu, The Netherlands) at 593 nm wavelength. We note that the dynamic range of the instrument was limited to read the dose amounts of 500 mg/kg for the BR extract and 50 mg/kg dose for silymarin administered to the rats daily, as described below. To compensate this deficiency, we performed the measurements in equivalent amounts obtained by scaling the administered doses up by 20 times, respectively. The readings from the mixtures containing the BR extract and silymarin were compared against the following standards: Gallic acid, Quercetin, Ascorbic acid, Rutin, Trolox, and BHT (2,6-di-tert-butyl-4-methyl phenol) [14].

### 2.4. Toxicity of the BR Extract

The toxicity of the BR extract was evaluated in normal healthy rats by subjecting them significantly to high doses of the extract. Rats were assigned equally into 3 groups, each with 6 males and 6 females, labeled as vehicle (10% Tween-20, 5 mL/kg) and 2 g/kg and 5 g/kg of rhizome extract preparation, respectively. The rats were deprived of food but not water prior to the dosing. Food was withheld for another 3-4 hours after the dosing. The animals were observed at 30 min and 2, 4, 8, 24, and 48 hours after the oral administration to detect the onset of clinical or toxicological symptoms. The animals were sacrificed on day 15. Through the jugular vein, blood was collected directly at the time of the sacrifice. Histological Prothrombin time and serum biochemical parameters were determined following the standard methods [16].

### 2.5. Treatment Groups and Experiments

A set of experiments were carried out to test the therapeutic effects of the BR extract on liver cirrhosis. For this purpose, thirty male rats were acquired and randomly divided into 5 groups where each consisted of 6 rats. The experiments lasted for 8 weeks, and all of the rats were kept alive during this timeframe. Classifications of the groups were as follows.

Group 1 served as the normal control group. The rats in this group were administered orally with 10% Tween-20 (5 mL/kg) daily and injected intraperitoneally (IP) with sterile distilled water (1 mL/kg) thrice weekly.

The rats in the remaining *Groups 2–5* were exposed to Thioacetamide (TAA) toxicity to induce cirrhosis in their livers. Constant exposure with this amount of TAA induces changes in liver pathology from both biological and morphological aspects comparable to the etiology of cirrhosis seen in humans [17] and therefore used very often as a preferred model in experimental studies of liver cirrhosis. Highest grade of TAA was purchased in crystal form from Chemolab Supplies, (Sigma-Aldrich, USA). The crystals were diluted in sterile distilled water and stirred well until all fully dissolved to prepare a stock solution of 5 g/L. TAA was injected IP three times a week at a dose of (200 mg/kg/mL in distilled water) [18].

Group 2 served as the cirrhosis control group with cirrhotic rats injected IP with TAA three times a week at a dose of (200 mg/kg/mL in distilled water) and oral delivery of 10% Tween 20 (5 mL/kg) daily.

Group 3 was the silymarin-treated group. The cirrhotic rats in this group were administered orally with silymarin (50 mg/kg) daily. Silymarin (International Laboratory, USA) is a standard drug and was prepared by dissolving in 10% Tween 20 [19].

Groups 4 and 5 were the treatment groups, where the cirrhotic rats were administered orally with the BR extract at respectively 250 mg/kg and 500 mg/kg doses daily.

We rationalized that the above protocol of applying treatment with silymarin or the BR extract in parallel after inducing cirrhosis using TAA injection was clinically equivalent to instituting the therapy as soon as the onset of the cirrhosis was diagnosed. In this regard, the treatment is preventive since it slows down the progression of the cirrhosis and protective since the liver is protected from further deterioration.

After 8 weeks, each rat was made to fast for 24 hours after the last treatment and then perfused under Ketamine (30 mg/kg, 100 mg/mL) and Xylazil (3 mg/kg, 100 mg/mL) anesthesia [20]. Through the jugular vein, blood was withdrawn and collected for prothrombin time and biochemical examinations. After the perfusion, the liver was excised and washed in ice-cold normal saline, blotted in filter paper, weighed and carefully inspected for the presence of any gross pathology. The liver tissues were further assessed as described below.

### 2.6. Postmortem Liver Tissue Analysis

For the histopathological analysis, the liver specimens were fixed in 10% buffered formaldehyde, processed by automated tissue processing machine, and then embedded in paraffin wax. Sections were prepared in 5 *μ*m thicknesses, stained with hematoxylin-eosin (H&E), and examined under the light microscope.

For determining the normality of the hepatocytes, the number of normal cells was counted at the center of the cirrhotic area as well as the normal areas adjacent to both sides of the cirrhotic area using a light microscope with an oil immersion objective (×40) covering 0.15 mm^2^ [21]. Percentage of the normal cells was calculated by using the formula: %Normal cells = [(Normal cells/(Normal + apoptotic cells) × 100].

### 2.7. Evaluation of Cellular Damage

Malondialdehyde (MDA) is a natural product of lipid peroxidation after cellular injury, and used as an indicator of cellular oxidative stress [22]. Superoxide dismutase enzyme (SOD) plays crucial role in defense mechanisms governing the anti-oxidant activities and hence in prevention of diseases linked to oxidative stress [23]. To examine the actions of the BR extract on the levels of MDA and SOD in the livers of the rats in all experimental Groups 1–5, the liver tissues were extracted, washed in saline, homogenized (10% w/v) in 50 mM cold potassium phosphate buffer (pH 7.4) by using tephlon homogenizer (Polytron, Heidolph RZR 1, Germany), and processed at 3500 rpm for 10 minutes at 4°C in a centrifuge (Heraeus, Germany). The MDA level was measured from the supernatant using thiobarbituric acid as the lipid peroxidation marker [24]. Similarly, the SOD activity was assessed based on a method described in [25].

### 2.8. Biochemical Analysis

On sacrifice, blood samples of the rats were collected through the jugular vein into tubes with sodium citrate for determining prothrombin time or into gel-activated tubes for the assessment of lipid profile and biochemical markers such as alkaline phosphatase (AP), alanine aminotransferase (ALT), aspartate aminotransferase (AST), lactate dehydrogenase (LDH), total protein, albumen, and bilirubin. The gel-activated tubes were allowed to clot, centrifuged at 3000 rpm for 10 minutes at 4°C. The serum samples were used for measuring the liver biochemicals. The markers were spectrophotometrically assayed by standard-automated techniques using the equipment at the Central Diagnostic Laboratory of the Medical Centre of University Malaya.

### 2.9. Statistical Analysis

The measurements from the experimental Groups 1–5 were evaluated statistically, and the statistical differences between the groups were determined using one-way ANOVA followed by Tukey Post-Hoc test analysis using SPSS program (version 18, SPSS Inc., Chicago, IL, USA). A value of *P* < 0.05 was considered as an indicative of statistically significant difference between the measurements of the two compared groups. All readings and calculated values were reported as Mean ± SEM.

## 3. Results

### 3.1. Anti-Oxidant Property of the BR Extract

**Figure 1:**
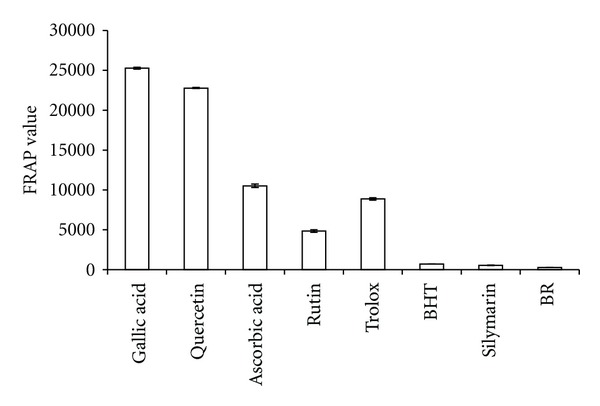
Antioxidant activity of the BR extract compared with the following standards: Gallic acid, Quercetin, Ascorbic acid, Rutin, Trolox, BHT as well as the standard drug Silymarin. Values were expressed as Mean ± SEM. Significant value was at *P* < 0.001.

As shown in Figure 1, the ferric reducing antioxidant power (FRAP) of 1 mg/mL of BR extract was measured as 288.9 ± 0.002 mmol/1 mg while the calibration curve equation was *y* = 0.0006 + 0.065, *R* ^2^ = 0.9976. The measured value was relatively lower than those of the standards Gallic acid, Quercetin, Ascorbic acid, Rutin, Trolox, and BHT, but comparable to the reference drug silymarin, which read 600.6 ± 0.003 mmol/0.1 mg. Silymarin contains several anti-oxidant compounds that play hepatoprotective roles. The BR extract may be playing similar role as it has free radical scavenging property and hence help the liver maintain its status quo.

### 3.2. Acute Toxicity Test

**Figure 2:**
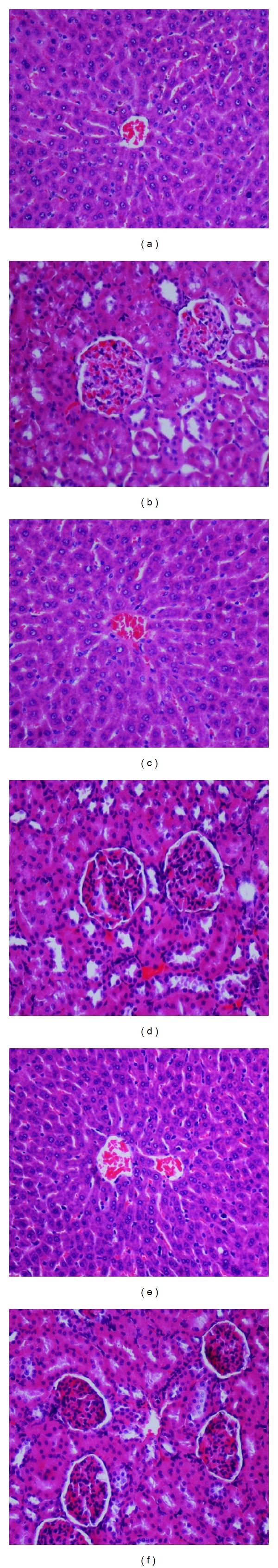
Examples of H&E-stained histological sections of livers (left column) and kidneys (right column) obtained from the rats in the acute toxicity test. Rat with the liver and kidney shown in (a) and (b) was treated with 5 mL/kg vehicle (10% Tween 20). Rat with the liver and kidney shown in (c) and (d) was treated with 2 g/kg (5 mL/kg) dose of the BR extract. Rat with the liver and kidney shown in (e) and (f) was treated with 5 g/kg (5 mL/kg) dose of the BR extract. There was no significant difference in the structures of the liver and kidney between the treatment and control groups.

All the rats in the acute toxicity test remained alive and did not manifest mortality or any visible signs of toxicity throughout the 15-days-long study at the high doses of the BR extract 2 g/kg and 5 g/kg that they were subjected to. The physical observations indicated no signs of changes in their skins and furs, eyes and mucus membranes, behavior patterns, tremors, salivations, diarrhea occurrences, and sleeps. The body weight of the treated male and female rats increased gradually but were not significantly different as compared to those of the control rats. Gross necropsy findings did not reveal visible changes in any of the organs. The clinical observations were that serum biochemical measurements reflected the functional status of normal kidney and liver, and the histopathological evaluations of the kidney and the liver tissues all together revealed that there were no significant differences between the control and test groups, as shown by the quantitative data in Tables 1 and 2, and qualitative data in Figure 2. 

### 3.3. Effects of the BR Extract on Liver Cirrhosis

#### 3.3.1. Body Weight and Liver Index

The total body of each rat was weighted prior to the sacrifice. Similarly, the liver was weighted after being excised (Table 3). The control rats in Groups 1 followed natural growth pattern and attained normal weight gains from about 200 g to 347 g in 8 weeks. The injection of TAA made the rats hepatotoxic (Group 2) and suffer growth retardation as they weighted significantly less (mean = 217 g) than those measured from the other groups. When the body weights were factored in, the cirrhotic rats in Group 2 had the highest liver index (mean = 5.27). The rats in the silymarin and high dose (500 mg/kg) BR treatments in Groups 3 and 5, respectively, attained weights as equivalent as the normal rats in Group 1. The rats in the low-dose (250 mg/kg) BR treatment Group 4 had better weight gain than those in Group 2, but not as much as those attained in Groups 3 and 5.

These findings implied that the outcome of the treatment was susceptible to the administered dose amount, but the BR extract at this high-dose appeared to be optimal since it was as effective as silymarin in counteracting the progression of cirrhosis. In light of this, we suggest administering the 500 mg/kg dose of the BR extract in strategizing any treatment plan targeting to offset cirrhosis in the future experimental studies with translational focus.

#### 3.3.2. Gross Anatomy and Histopathology

**Figure 3:**
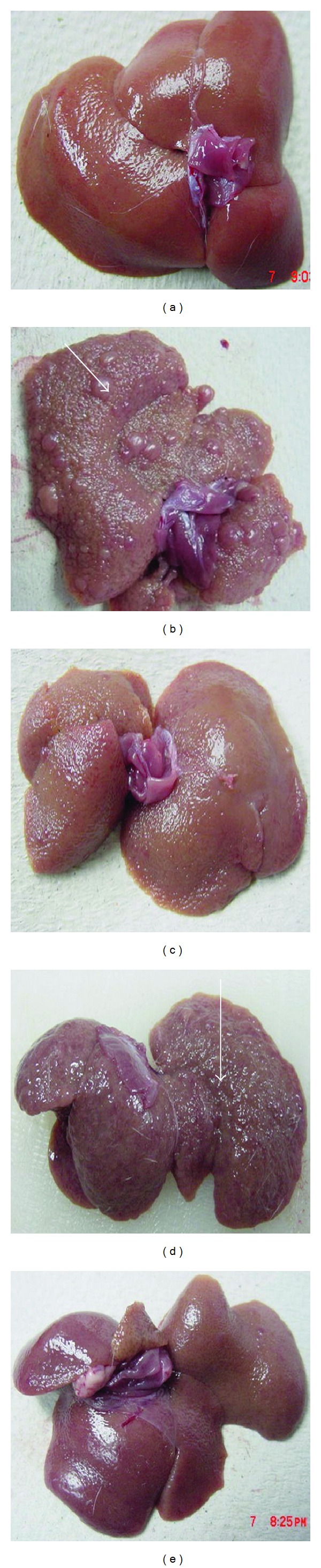
Example images showing macroscopic appearances of the livers sampled from rats in different experimental groups. (a) The liver of a control rat exhibiting regular smooth surface. (b) The liver of a hepatotoxic rat depicting numerous irregular whitish micro- and macronodular on its surface and a large area of ductular cholangiocellular proliferation (arrow) embedded within fibrosis. (c) The liver of a hepatoprotective rat treated with silymarin showing normal smooth surface. (d) The liver of a rat treated with 250 mg/kg of the BR extract illustrating nearly smooth surface with fewer granules (arrow head). (e) The liver of a rat treated with 500 mg/kg of the BR extract having normal smooth surface.

**Figure 4:**
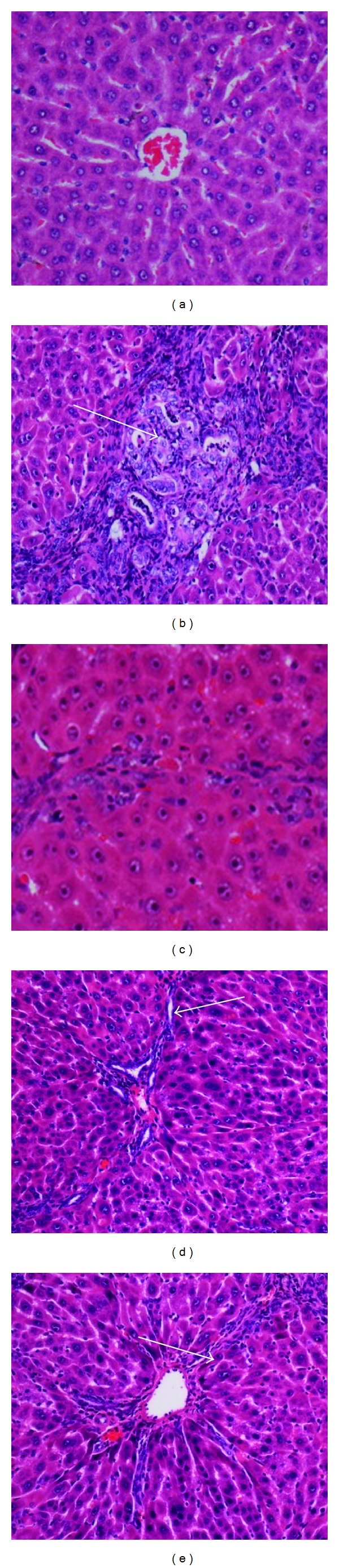
Example histopathological sections of livers sampled from rats in different experimental groups. (a) Normal histological structure and architecture were seen in livers of the control rats. (b) Severe structural damage, formation of pseudolobules with thick fibrotic septa and necrotic areas were present in the liver of the hepatotoxic rat. (c) Mild inflammation but no fibrotic septa was depicted in the liver of the hepatoprotective rat treated with silymarin. (d) Partially preserved hepatocyte and architecture with small area of necrosis and fibrotic septa existed in the liver of the rat treated with 250 mg/kg of the BR extract. (e) Partially preserved hepatocyte and architecture with small areas of mild necrosis were observed in the liver of the rat treated with 500 mg/kg of the BR extract. (H&E stain original magnification 20x).

Figures 3 and 4, respectively, depict the gross appearances and the H&E-stained sections of the example liver samples from the experimental Groups 1–5. Grossly, the livers from the control rats in Group 1 (Figure 3(a)) appeared in reddish color, had smooth surfaces, and did not show any sign of nodules. The histological examination (Figure 4(a)) showed normal liver architecture with normal plates of hepatocytes separated by sinusoidal capillaries and central vein. In cirrhotic Group 2, the liver appeared congested with numerous micro- and macronodules (Figure 3(b)), lost its normal architecture by the presence of regenerating nodules that were separated by fibrous septae extending from the central vein to portal triad (Figure 4(b)). In addition, the fibrous septae were accompanied by severe proliferation of bile duct and heavy invasion of inflammatory cells. The cirrhotic nodules showed thick purple-colored bundles of collagen fibers. The livers of the reference control silymarin Group 3 (Figures 3(c) and 4(c)) and the high doses of BR extract (Figures 3(e) and 4(e)) showed a relatively minor micronodules, a lesser amount of fibrous septae development and expansion and an increase in the extension of normal hepatic parenchyma compared to those from the reference Group 3. In contrast, the livers of the low-dose BR Group 4 were occupied by lesser macronodules and lesser fibrotic nodules than those of the reference Group 3, but the improvements were not as much as those seen in Groups 3 or 5. These results based on the visual evaluations provided further independent confirmation that the applied BR extract was effectively protecting the liver against the progression of cirrhosis.

#### 3.3.3. Cell Loss and Survival

**Figure 5:**
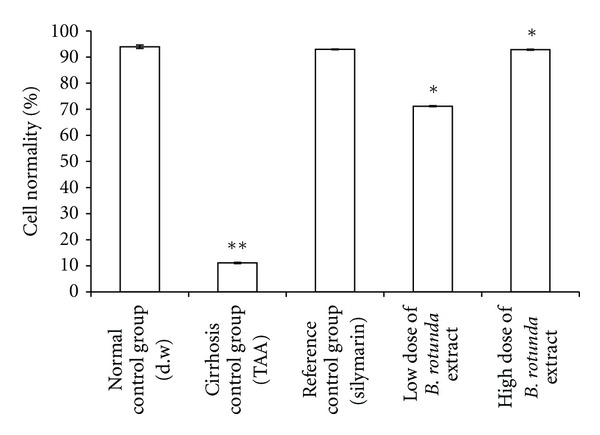
Effect of BR ethanol extract on % cell normality. Data are expressed as mean ± SEM. Means among groups (*n* = 6 rate/group) show significant difference, *P < 0.001 compared to cirrhosis control group and **P < 0.001 compared to normal control group.

The results concerning the normality of the hepatocytes were illustrated in Figure 5 for the rats in Groups 1–5. According to the data, the administration of TAA has observed to significantly decrease the number of normal liver cells from about 94% measured from the livers of the normal rats in Group 1 to about 11% measured from the livers of the cirrhotic rats in Group 2. Hepatocytic fatty degeneration was also present. In the low dose BR (250 mg/kg) treatment Group 4, the population of the normal cells was higher, about 71%. But, the treatment with the high dose BR maintained much higher number of normal cells, about 93%, which was nearly equal to those obtained from the silymarin-treated rats in Group 3 and comparable in the same manner to the normal rats in Group 1.

**Figure 6:**
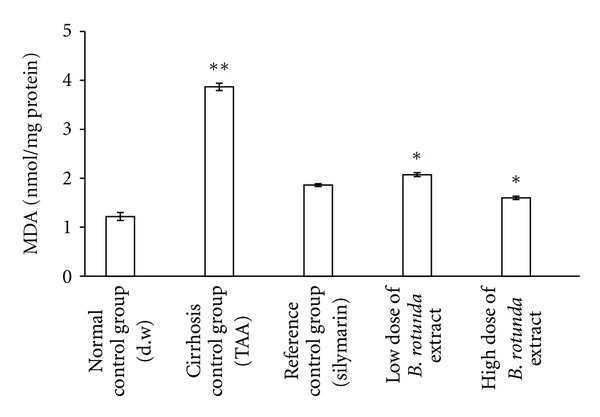
Effect of the BR extract on the level of MDA in the liver tissue. Data were expressed as Mean ± SEM. Means between the silymarin treated Group 3, low-dose BR-treated Group 4, and high-dose BR-treated Group 5 had significant differences when compared with the cirrhosis control Group 2 with *P < 0.001  and compared with the normal control Group 1 with **P < 0.001.

**Figure 7:**
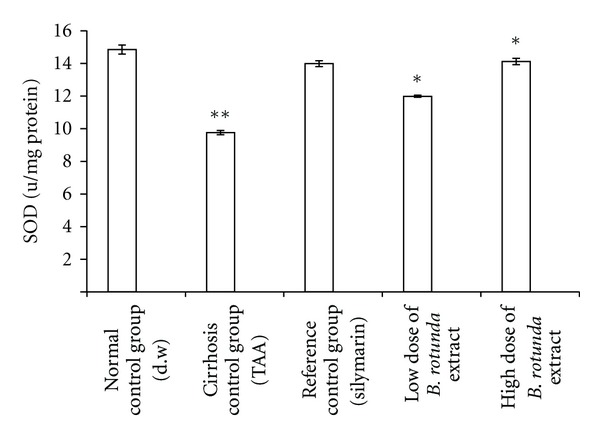
Effect of BR ethanol group on SOD level in the liver tissue. Data are expressed as mean ± SEM. Means among groups (*n* = 6 rate/group) show significant difference, *P < 0.001 compared to cirrhosis control group, and **P < 0.001 compared to normal control group.

The loss of hepatocytes in the livers of the cirrhotic rats was probed indirectly via lipid peroxidation with MDA and anti-oxidant enzymatic activity with SOD, and the results were plotted in Figures 6 and 7. The MDA level reads relatively high value of 3.87 ± 0.08 nmol/mg protein in the cirrhosis control group when compared with the reading from the normal group 1.22 ± 0.08 nmol/mg protein. The SOD readings followed this trend but inversely, meaning that the cirrhotic rats in Group 2 had lower value of 9.80 ± 0.13 u/mg protein than 14.89 ± 0.28 *μ*/mg protein from the normals in Group 1. These results indicated the presence of severely damaged cells in the cirrhotic livers. Treating the cirrhotic rats with the BR extract has significantly helped the survival of the hepatocytes as indicated by the reduced MDA and increased SOD levels in both the low- and high-dose groups, but the effect was more pronounced in the latter group. In the low- and high-dose groups, the MDA reads 2.08 ± 0.04 nmol/mg protein versus 1.60 ± 0.03 nmol/mg protein, and SOD reads 12.03 ± 0.06 u/mg protein versus 14.17 ± 0.19 u/mg protein, respectively. The MDA and SOD readings from the normal group were 1.22 ± 0.08 nmol/mg protein and 14.89 ± 0.28 u/mg protein, respectively and the corresponding values for the silymarin treated-group were 1.86 ± 0.03 nmol/mg protein and 14.03 ± 0.18 u/mg protein, respectively. These results collectively suggested that the BR treatment provided a host environment favorable for both preventing and protecting the hepatocytes from further damage.

#### 3.3.4. Liver Markers and Lipid Profiles

Liver function of each rat was measured by determining the plasma levels of specific liver enzymes and lipid profile, and the results were presented in Tables 4, 5, and 6. According to the data in Tables 4 and 5, the TAA-induced liver damage significantly elevated the levels of specific liver enzymes AP, ALT, AST, LDH, bilirubin and prothrombin time ratio (*P* < 0.001) and significantly declined the protein and albumin levels in the cirrhotic rats of Group 2 as compared with those measured from all the other groups. Similarly, the lipid profiles in the cirrhotic rats altered significantly such that the cholesterol, LDL, and triglycerides levels were higher, and the HDL level was lower (Table 6). The high-dose BR (500 mg/kg) treatment Group 5 resulted in the readings on the biochemical markers that were comparable to those of the control Group 1 and the silymarin-treated (50 mg/kg) Group 3, and better than the readings obtained from the treatment with the low-dose BR (250 mg/kg) Group 4. These data further supported the qualitative gross anatomical, histopathological findings, the quantitative cell counts, presented above, and demonstrated that the effects of the toxicity induced by TAA on the liver function can effectively be counterbalanced by the positive effects of the BR extract treatment, but in a dose-dependent manner.

## 4. Discussion

Liver cirrhosis has become a serious public health problem because of the broader use of prescription drugs with side effects in modern life or the substance abuse. Consequently, the current research has focused on understanding the underlying metabolisms and subsequently finding new therapeutic solutions to interrupt the signaling pathways and minimize the damages inflicted on the liver [26]. Beyond the strategies with synthetic pharmacology, the search also pursues alternative approaches that rely on natural products. Especially, it targets those plants in the folk medicine with known history or demonstrated potential of positive effects against the diseases of the liver or other organs [27]. To aid these efforts, in this study, we examined the potential of ethanol-based extract from the plant BR as a promising therapeutic agent for treating liver cirrhosis.

In phase 2 of our study, we tested the toxicity of the BR extract. Our data in Figure 2 and Tables 1 and 2 showed that the extract at high doses caused no significant pathological abnormalities in the liver and kidney, and the clinical biochemistry readings remained within the normal range. These results were in agreement with the previous reports on the safety of consuming the BR extract [28].

In the next set of experiments, we examined the influence of the BR extract on the course of the cirrhosis development. The cirrhotic condition was induced by a prolonged exposure to TAA. Manipulating the amount of TAA dose produces different grades of liver damage that may range from the parenchymal cell necrosis and liver cell proliferation to the production of pseudolobules and nodular cirrhosis, [29]. In this study, we have chosen 200 mg/kg dose-administered IP for 2 months because this protocol was reported to yield etiology similar to the human cirrhosis in terms of anatomical, structural, functional, and architectural tissue characteristics as well as the readings on the common biochemical markers [30]. The same was again reconfirmed collectively by the qualitative ex vivo visualization of the nodules in Figures 3 and 4, the quantitative data on the lower body weights of the cirrhotic rats in Table 3, the biochemical imbalances in the liver markers in Tables 4 and 5, and altered lipid profiles in Table 6. Therefore, our experimental rat model of cirrhosis was suitable for testing the efficacy of any applied preclinical therapy with a clinical translation in focus.

The TAA action in the development of cirrhosis was suggested to be multifaceted and to involve multiple mechanisms [19]. For example, TAA interferes with the RNA transference from nucleus to cytoplasm via its metabolite thioacetamide-S-oxide (TASO_2_). Concerning the hepatocytes, a compromise in the RNA transfer leads to hepatic cell death via the processes of apoptosis and necrosis [31]. Quantitative histopathological analysis indicated the presence of severe hepatocellular loss since the percentage of the viable cells were substantially lower 11% in the cirrhotic rats in Group2 than 94% in normals in Group 1 (Figure 5). Lipid peroxidation is also a common event in a toxic phenomenon and causes cell death due to the degradation of membrane lipids [32]. Through another pathway, the TAA toxicity contributes to the liver damage by stimulating the production of excessive reactive oxygen species [33]. This effect was recorded in Figures 6 and 7. The data indicated that the hepatocytes were under oxidative stress, lipid peroxidation was prevalent as reflected by the larger MDA readings, and the anti-oxidant defense mechanism was failed as hinted by the attenuated SOD readings.

The cirrhotic livers responded favorably to the treatment with the BR extract at both doses (250 mg/kg and 500 mg/kg) and the reference extract silymarin at the daily dose of 50 mg/kg. Each of the treatment regimens unequivocally produced significant improvements in the anatomical, structural, functional, and architectural signatures of the otherwise cirrhotic livers. The efficacy in protecting the liver was however marginally better with the higher BR dose, and encouragingly very close to that of silymarin. Nearly identical responses between the high-dose BR and silymarin treatments can be explained by the anti-oxidant powers of the two extracts, as asserted by the FRAP measurements in Figure 1.

In the treated rats, the rise of the serum levels of ALT, AST, AP, LDH, and bilirubin and the decline in the levels of albumin and total protein were prevented. The trends in restoring the balance in serum chemicals may be attributed to the capacity of the BR extract to regulate the hepatic antioxidant status or to directly participate in the radical scavenging process [34]. In the TAA metabolism, anti-oxidants work against oxidative stress by scavenging the byproduct TASO_2_ and, thereby, reducing the magnitude of the impact on the liver. Phenol compounds are very effective anti-oxidants with strong free radical-scavenging abilities [35]. Flavonoids are polyphenols and used in treating many diseases including liver cirrhosis. Flavonoids (Kaempferol and Quercetin) are present in the plant BR and therefore likely be responsible for the membrane stabilizing activities demonstrated by the observed reductions in the serum levels of the liver enzymes. The lowered bilirubin levels meant the presence of more stable erythrocyte plasma membranes. This consequently implied that the BR extract stabilized the hepatocyte membranes and hence interrupted the release of the liver enzymes into the blood [36]. The inhibitory effect of BR extract on the lipid peroxidation of the macromolecules in the membrane can again be credited to the scavenging effect of the flavonoid content of the plant [37].

## 5. Conclusions

The progression of the liver cirrhosis induced by TAA in rats can be inhibited using ethanol-based BR extract. Specifically, this natural extract has the power to protect the liver by preventing the cascade of harmful events in liver cirrhosis. The effects are comparable to those of silymarin (50 mg/kg) when the corresponding daily dose was 500 mg/kg. The capability of the BR extract to preserve the liver status quo of property, structure, and function against toxic exposure is encouraging and warrants further studies. The significance of its pharmacologic potential in successfully treating liver cirrhosis can be explored by mapping the molecular pathways of its action in the future.

## Figures and Tables

**Table 1:** Renal function measured from the acute toxicity test of the BR extract on rats.

Dose	Sodium (mmol/L)	Potassium (mmol/L)	Chloride (mmol/L)	Urea (mmol/L)	Creatinine (*μ*mol/L)
Vehicle (5 mL/kg)	138.25 ± 0.45	5.03 ± 0.19	104.03 ± 0.15	5.63 ± 0.41	50.18 ± 1.34
LD (2 g/kg)	137.65 ± 0.43	5.21 ± 0.16	102.61 ± 1.22	4.96 ± 0.43	48.97 ± 0.81
HD (5 g/kg)	137.21 ± 0.51	4.89 ± 0.15	102.67 ± 0.76	5.93 ± 0.39	48.60 ± 1.80

The values were expressed as mean ± S.E.M. There were no significant differences between the three groups. Significant value was at *P* < 0.05.

**Table 2:** Liver function measured from the acute toxicity test of the BR extract on rats.

Dose	Total protein (g/L)	Albumin (g/L)	TB (*μ*mol/L)	AP (IU/L)	ALT (IU/L)	AST (IU/L)	GGT (IU/L)
Vehicle (5 mL/kg)	71.37 ± 1.44	11.36 ± 0.53	1.91 ± 0.17	134.78 ± 9.57	53.05 ± 3.27	153.65 ± 9.35	4.91 ± 0.93
LD (2 g/kg)	71.47 ± 0.52	11.61 ± 0.34	2.18 ± 0.16	133.37 ± 8.63	51.90 ± 1.33	156.07 ± 3.56	5.00 ± 1.23
HD (5 g/kg)	71.81 ± 1.03	11.72 ± 0.16	1.88 ± 0.21	135.13 ± 6.52	52.27 ± 3.25	155.00 ± 5.35	5.32 ± 1.07

TB: total bilirubin; AP: alkaline phosphatase; ALT: alanine aminotransferase; AST: aspartate aminotransferase; GGT: gamma-glutamyl transferase. The values were expressed as mean ± S.E.M. There were no significant differences between the three groups. Significant value was at *P* < 0.05.

**Table 3:** Liver index measurements from the rats at the end of the 8-week study.

Treatment	Body weight (gm)	Liver weight (gm)	Liver index (LW/BW %)
Group 1 (normal rats)	347 ± 5.04	9.70 ± 0.16	2.79 ± 0.21
Group 2 (rats with cirrhosis)	183 ± 2.32	9.44 ± 0.60	5.18 ± 0.06**
Group 3 (silymarin-treated rats)	347 ± 3.58	10.28 ± 0.66	2.97 ± 0.35*
Group 4 (treatment with the BR extract at 250 mg/kg dose)	255 ± 2.91	10.03 ± 0.46	3.93 ± 0.44*
Group 5 (treatment with the BR extract at 500 mg/kg dose)	376 ± 5.67	9.71 ± 0.71	2.59 ± 0.36*

Data were expressed as Mean ± SEM. Means between the silymarin-treated Group 3, low-dose BR-treated Group 4, and high-dose BR-treated Group 5 had significant differences when compared with the cirrhosis control Group 2 with *P < 0.001 and compared with the normal control Group 1 with **P < 0.001.

**Table 4:** Effect of BR ethanol extract on plasma levels of specific liver enzymes at the end of the 8-week study.

Treatment	AP IU/L	ALT IU/L	AST IU/L	LDH IU/L
Group 1 (normal rats)	79.28 ± 0.58	30.57 ± 1.51	70.35 ± 0.21	490.97 ± 3.90
Group 2 (rats with cirrhosis)	270.50 ± 4.88**	150.42 ± 2.60**	250.88 ± 2.99**	991.72 ± 5.01**
Group 3 (silymarin-treated rats)	83.85 ± 1.06	31.62 ± 0.63	70.35 ± 0.43	546.33 ± 10.46
Group 4 (treatment with the BR extract at 250 mg/kg dose)	110.22 ± 0.49*	91.02 ± 1.61*	106.00 ± 2.59*	792.00 ± 3.51*
Group 5 (treatment with the BR extract at 500 mg/kg dose)	86.13 ± 0.56*	33.42 ± 0.56*	71.10 ± 0.78*	574.00 ± 5.34*

AP: alkaline phosphatase; ALT: alanine transferase; AST: aspartate transferase; LDH lactate dehydrogenase. Means between the silymarin-treated Group 3, low-dose BR-treated Group 4, and high-dose BR-treated Group 5 had significant differences when compared with the cirrhosis control Group 2 with *P < 0.001 and compared with the normal control Group 1 with **P < 0.001.

**Table 5:** Effect of Br ethanol extract on serum protein, albumen, and globulin levels and prothrombin time ratio at the end of the 8-week study.

Treatment	Protein g/L	Albumen g/L	Bilirubin umol/L	Prothrombin time ratio
Group 1 (normal rats)	76.58 ± 0.70	31.93 ± 0.64	1.20 ± 0.06	1.02 ± 0.006
Group 2 (rats with cirrhosis)	59.92 ± 1.14**	11.22 ± 0.33**	4.98 ± 0.12**	1.38 ± 0.024**
Group 3 (silymarin-treated rats)	75.82 ± 0.70	32.38 ± 0.88	1.28 ± 0.07	1.02 ± 0.005
Group 4 (treatment with the BR extract at 250 mg/kg dose)	63.37 ± 1.20	20.20 ± 0.20*	2.85 ± 0.10*	1.28 ± 0.009*
Group 5 (treatment with the BR extract at 500 mg/kg dose)	75.10 ± 1.07*	29.38 ± 0.28*	1.68 ± 0.05*	1.03 ± 0.002*

Means between the silymarin-treated Group 3, low-dose BR-treated Group 4, and high-dose BR-treated Group 5 had significant differences when compared with the cirrhosis control Group 2 with *P < 0.001 and compared with the normal control Group 1 with **P < 0.001.

**Table 6:** Effect of BR ethanol extract on serum lipid profiles at the end of the 8-week study.

Treatment	Cholesterol mmol/L	HDL mmol/L	LDL mmol/L	Triglycerides mmol/L
Group 1 (normal rats)	1.42 ± 0.05	1.13 ± 0.04	0.13 ± 0.02	1.27 ± 0.03
Group 2 (rats with cirrhosis)	3.22 ± 0.06**	0.53 ± 0.04**	2.50 ± 0.10**	2.83 ± 0.07**
Group 3 (Silymarin-treated rats)	1.80 ± 0.04	1.32 ± 0.04	0.33 ± 0.03	1.45 ± 0.04
Group 4 (treatment with the BR extract at 250 mg/kg dose)	2.00 ± 0.06*	0.62 ± 0.07	1.22 ± 0.07*	1.78 ± 0.03*
Group 5 (treatment with the BR extract at 500 mg/kg dose)	1.57 ± 0.05*	1.17 ± 0.05*	0.23 ± 0.02*	1.42 ± 0.05*

Means between the silymarin-treated Group 3, low-dose BR-treated Group 4, and high-dose BR-treated Group 5 had significant differences when compared with the cirrhosis control Group 2 with *P < 0.001 and compared with the normal control Group 1 with **P < 0.001.

## References

[1] Raison CL, Demetrashvili M, Capuron L, Miller AH (2005). Neuropsychiatric adverse effects of interferon-*α*: recognition and management. *CNS Drugs*.

[2] Yang H, Sung SH, Kim YC (2005). Two new hepatoprotective stilbene glycosides from Acer mono leaves. *Journal of Natural Products*.

[3] Wang N, Li P, Wang Y (2008). Hepatoprotective effect of *Hypericum japonicum* extract and its fractions. *Journal of Ethnopharmacology*.

[4] El-Abhar HS, Hammad LNA, Gawad HSA (2008). Modulating effect of ginger extract on rats with ulcerative colitis. *Journal of Ethnopharmacology*.

[5] Shindo K, Kato M, Kinoshita A, Kobayashi A, Koike Y (2006). Analysis of antioxidant activities contained in the *Boesenbergia pandurata* Schult. rhizome. *Bioscience, Biotechnology and Biochemistry*.

[6] Voravuthikunchai SP, Phongpaichit S, Subhadhirasakul S (2005). Evaluation of antibacterial activities of medicinal plants widely used among AIDS patients in Thailand. *Pharmaceutical Biology*.

[7] Kirana C, Jones GP, Record IR, McIntosh GH (2007). Anticancer properties of panduratin A isolated from *Boesenbergia pandurata* (Zingiberaceae). *Journal of Natural Medicines*.

[8] Stevenson PC, Veitch NC, Simmonds MSJ (2007). Polyoxygenated cyclohexane derivatives and other constituents from *Kaempferia rotunda* L.. *Phytochemistry*.

[9] Othman R, Kiat TS, Khalid N (2008). Docking of noncompetitive inhibitors into dengue virus type 2 protease: understanding the interactions with allosteric binding sites. *Journal of Chemical Information and Modeling*.

[10] Rho HS, Ghimeray AK, Yoo DS (2011). Kaempferol and kaempferol rhamnosides with depigmenting and anti-inflammatory properties. *Molecules*.

[11] Ching AYL, Tang SW, Sukari MA, Lian GEC, Rahmani M, Khalid K (2007). Characterization of flavonoid derivatives from *Boesenbergia rotunda* (L.). *The Malaysian Journal of Analytical Sciences*.

[12] Alshawsh MA, Abdulla MA, Ismail S, Amin ZA (2011). Hepatoprotective effects of *Orthosiphon stamineus* extract on thioacetamide-induced liver cirrhosis in rats. *Evidence-Based Complementary and Alternative Medicine*.

[13] Pradhan SC, Girish C (2006). Hepatoprotective herbal drug, silymarin from experimental pharmacology to clinical medicine. *The Indian Journal of Medical Research*.

[14] Pliny the Elder Historia Naturalis 77 A.D..

[15] Jing LJ, Mohamed M, Rahmat A, Bakar MFA (2010). Phytochemicals, antioxidant properties and anticancer investigations of the different parts of several gingers species (*Boesenbergia rotunda*, *Boesenbergia pulchella* var attenuata and *Boesenbergia armeniaca*). *Journal of Medicinal Plant Research*.

[16] Mahmood AA, Mariod AA, Abdelwahab SI, Ismail S, Al-Bayaty F (2010). Potential activity of ethanolic extract of *Boesenbergia rotunda* (L.) rhizomes extract in accelerating wound healing in rats. *Journal of Medicinal Plants Research*.

[17] Beale G, Chattopadhyay D, Gray J (2008). AFP, PIVKAII, GP3, SCCA-1 and follisatin as surveillance biomarkers for hepatocellular cancer in non-alcoholic and alcoholic fatty liver disease. *BMC Cancer*.

[18] Aydin AF, Küskü-Kiraz Z, Doğru-Abbasoğlu S, Güllüoğlu M, Uysal M, Koçak-Toker N (2010). Effect of carnosine against thioacetamide-induced liver cirrhosis in rat. *Peptides*.

[19] Ahmad A, Pillai KK, Najmi AK, Ahmad SJ, Pal SN, Balani DK (2002). Evaluation of hepatoprotective potential of jigrine post-treatment against thioacetamide induced hepatic damage. *Journal of Ethnopharmacology*.

[20] Fatemi F, Allameh A, Khalafi H, Ashrafihelan J (2010). Hepatoprotective effects of *γ*-irradiated caraway essential oils in experimental sepsis. *Applied Radiation and Isotopes*.

[21] Al Bayaty F, Abdulla M, Abu Hassan MI, Masud MI (2010). Wound healing potential by hyaluronate gel in streptozotocin-induced diabetic rats. *Scientific Research and Essays*.

[22] Dash DK, Yeligar VC, Nayak SS (2007). Evaluation of hepatoprotective and antioxidant activity of *Ichnocarpus frutescens* (Linn.) R. Br. on paracetamol-induced hepatotoxicity in rats. *Tropical Journal of Pharmaceutical Research*.

[23] Ji LL (2008). Modulation of skeletal muscle antioxidant defense by exercise: role of redox signaling. *Free Radical Biology and Medicine*.

[24] Wang H, Wei W, Wang NP (2005). Melatonin ameliorates carbon tetrachloride-induced hepatic fibrogenesis in rats via inhibition of oxidative stress. *Life Sciences*.

[25] Guo W, Adachi T, Matsui R (2003). Quantitative assessment of tyrosine nitration of manganese superoxide dismutase in angiotensin II-infused rat kidney. *American Journal of Physiology*.

[26] Daly AK, Donaldson PT, Bhatnagar P (2009). HLA-B5701 genotype is a major determinant of drug-induced liver injury due to flucloxacillin. *Nature Genetics*.

[27] Khanna D, Sethi G, Ahn KS (2007). Natural products as a gold mine for arthritis treatment. *Current Opinion in Pharmacology*.

[28] Mahmood AA, Mariod AA, Abdelwahab SI, Ismail S, Al-Bayaty F (2010). Potential activity of ethanolic extract of *Boesenbergia rotunda* (L.) rhizomes extract in accelerating wound healing in rats. *Journal of Medicinal Plants Research*.

[29] Sadasivan S, Latha PG, Sasikumar JM, Rajashekaran S, Shyamal S, Shine VJ (2006). Hepatoprotective studies on *Hedyotis corymbosa* (L.) Lam.. *Journal of Ethnopharmacology*.

[30] Plonné D, Schulze HP, Kahlert U (2001). Postnatal development of hepatocellular apolipoprotein B assembly and secretion in the rat. *Journal of Lipid Research*.

[31] Chen LH, Hsu CY, Weng CF (2006). Involvement of P53 and Bax/Bad triggering apoptosis in thioacetamide-induced hepatic epithelial cells. *World Journal of Gastroenterology*.

[32] Yang Y, Sharma R, Sharma A, Awasthi S, Awasthi YC (2003). Lipid peroxidation and cell cycle signaling: 4-Hydroxynonenal, a key molecule in stress mediated signaling. *Acta Biochimica Polonica*.

[33] Sarkar K, Sil PC (2006). A 43 kDa protein from the herb *Cajanus indicus* L. protects thioacetamide induced cytotoxicity in hepatocytes. *Toxicology in Vitro*.

[34] Ajith TA, Hema U, Aswathy MS (2007). *Zingiber officinale* Roscoe prevents acetaminophen-induced acute hepatotoxicity by enhancing hepatic antioxidant status. *Food and Chemical Toxicology*.

[35] Arts ICW, Hollman PCH (2005). Polyphenols and disease risk in epidemiologic studies. *The American Journal of Clinical Nutrition*.

[36] Tang X, Gao J, Wang Y (2006). Effective protection of *Terminalia catappa* L. leaves from damage induced by carbon tetrachloride in liver mitochondria. *The Journal of Nutritional Biochemistry*.

[37] Prince Vijeya Singh J, Selvendiran K, Mumtaz Banu S, Padmavathi R, Sakthisekaran D (2004). Protective role of Apigenin on the status of lipid peroxidation and antioxidant defense against hepatocarcinogenesis in Wistar albino rats. *Phytomedicine*.

